# Utility of MALDI-TOF MS for determination of species identity and blood meal sources of primary malaria vectors on the Kenyan coast

**DOI:** 10.12688/wellcomeopenres.18982.2

**Published:** 2024-06-04

**Authors:** Jonathan Karisa, Kelly Ominde, Mercy Tuwei, Brian Bartilol, Zedekiah Ondieki, Harun Musani, Caroline Wanjiku, Kioko Mwikali, Lawrence Babu, Martin Rono, Mumin Eminov, Charles Mbogo, Philip Bejon, Joseph Mwangangi, Maureen Laroche, Marta Maia

**Affiliations:** 1 Kenya Medical Research Institute, Wellcome Trust Research Programme, Kilifi, Kenya, 230-80108, Kenya; 2 The Open University, Milton Keynes, United Kingdom, Walton Hall, Kents Hill, Milton Keynes MK7 6AA, UK; 3 Pwani University, Kilifi, Kenya, 195-80108, Kenya; 4 Bruker Daltonik GmbH, Bremen, Germany; 5 University of Oxford, Centre for Global Health and Tropical Medicine, Oxford, UK, Oxford, UK; 6 The University of Texas Medical Branch -, Galveston National Laboratory 301 University Blvd, Texas, Galveston TX 77555-1019, USA

**Keywords:** MALDI-TOF MS, entomological surveillance, Anopheles, high-throughput, mass spectrometry, Kenya, Coast

## Abstract

**Background:**

Protein analysis using matrix-assisted laser desorption/ionisation time-of-flight mass-spectrometry (MALDI-TOF MS) represents a promising tool for entomological surveillance. In this study we tested the discriminative power of this tool for measuring species and blood meal source of main Afrotropical malaria vectors on the Kenyan coast.

**Methods:**

Mosquito collections were conducted along the coastal region of Kenya. MALDI-TOF MS spectra were obtained from each individual mosquito’s cephalothorax as well as the abdomens of blood-engorged mosquitoes. The same mosquitoes were also processed using gold standard tests: polymerase chain reaction (PCR) for species identification and enzyme linked immunosorbent assay (ELISA) for blood meal source identification.

**Results:**

Of the 2,332 mosquitoes subjected to MALDI-TOF MS, 85% (1,971/2,332) were considered for database creation and validation. There was an overall accuracy of 97.5% in the identification of members of the *An. gambiae* ( *An. gambiae*, 100%; *An. arabiensis*, 91.9%; *An. merus*, 97.5%; and *An. quadriannulatus*, 90.2%) and *An. funestus* ( *An. funestus*, 94.2%; *An. rivulorum*, 99.4%; and *An. leesoni*, 94.1%) complexes. Furthermore, MALDI-TOF MS also provided accurate (94.5% accuracy) identification of blood host sources across all mosquito species.

**Conclusions:**

This study provides further evidence of the discriminative power of MALDI-TOF MS to identify sibling species and blood meal source of Afrotropical malaria vectors, further supporting its utility in entomological surveillance. The low cost per sample (<0.2USD) and high throughput nature of the method represents a cost-effective alternative to molecular methods and could enable programs to increase the number of samples analysed and therefore improve the data generated from surveillance activities.

## Introduction

Globally, malaria has significantly declined in the last decades, however countries in sub-Saharan Africa (SSA) continue to endure the high morbidity and highest mortality rates. In 2020, around 241 million cases and 627,000 related deaths were reported globally, with over 90% of the deaths reported in Africa ^1^. The WHO Global Technical Strategy (GTS) 2016–2030 is not on track having missed the target of reducing malaria case incidence and mortality by at least 40% by 2020 ^2^. Sustainable vector control strategies capable of addressing current gaps and enabling malaria elimination in SSA will require the development of new malaria vector control tools and approaches and/or improvement of existing ones to malaria vector control, as well as improved vector surveillance systems ^3^. One of the pillars of the global vector control response is the enhancement of vector surveillance. In order to design appropriate and effective vector control strategies, it is imperative to understand the behaviour, biology, and ecology of local vectors. Over 500 *Anopheles* species have been described globally, with approximately 50 species incriminated in malaria transmission as either primary or complementary vectors ^4– 7^. In Sub-Saharan-Africa, *Anopheles gambiae* and *An. funestus* complexes dominate ^5, 6^; and consist of morphologically indistinguishable members but with distinct biting and resting behaviour. Understanding their composition, distribution, and behaviour including blood feeding patterns and preferred human and animal hosts would be fundamental in designing effective control strategies. In general entomological practice the field, morphologically identical mosquitoes are sorted into complexes using taxonomic keys ^8, 9^ and sibling species distinguished using molecular methods such as polymerase chain reaction (PCR) ^10– 12^. On the other hand, blood meal sources are commonly identified using enzyme linked immunosorbent assays (ELISA) ^13, 14^. Although PCR is highly sensitive, it is technically demanding, time-consuming and has a high cost per sample. Contrastingly, ELISA is cheaper, but also equally laborious and impeded by unavailability of antibodies and cross-reactions that sometimes produce spurious results that are difficult to interpret ^15^.

MALDI-TOF MS is a protein profiling technique with a variety of applications that include microbiology, and entomology. In particular, it has revolutionised clinical microbiology by providing an accurate, rapid, and inexpensive identification of microorganisms ^16^. Most recently, this tool has been shown to be able to identify mosquitoes sibling species ^17– 20^, blood meal sources ^19, 21, 22^, as well as pathogen infection ^23– 26^. The low-cost per sample, rapidity, and accuracy of MALDI-TOF MS makes it a reliable method for biotyping different mosquito parameters with the potential to exponentially increase the number of specimens analysed by surveillance programs.

This current study evaluated the utility of MALDI-TOF MS in determining the identity of sibling species of the *An. gambiae* and *An. funestus* complexes and associated blood meal sources. To the best of our knowledge, this is the first-time members of the *An. funestus* complex have been characterised using MALDI-TOF MS.

## Methods

### Study area

Mosquito samples were collected from 2019–2022 in three distinct ecological zones with different species composition viz., Kilifi, Taita Taveta and Kwale Counties of Kenya. The Coastal region of Kenya encounters bimodal rainfall; long rains happening between April and July and short rains between October and December with mean annual rainfall of 750 to 1,200 mm. The relative humidity ranging between 55 and 65% and mean annual temperature between 20 to 35°C. The altitude ranges between 0 and 400 meters above sea level. Both Kilifi and Kwale counties are inhabited mostly by the Mijikenda; whereas Taita Taveta is mainly inhabited by the Taita and Taveta ethnic groups; communities that rely largely on farming and fishing and that live houses made of mud, or coral rock and roofed using palm leaves ( *makuti*) ^27– 29^.

In Kilifi County, sampling was conducted in Garithe, Burangi, Marana, Mtondia and Sihu villages ^30, 31^. In Kwale, sampling was conducted between June and July 2021 in 12 villages *viz* Fihoni, Jego, Marigiza, Mangwei, Gazi, Madongoni, Kikwezani, Kiwegu, Kidomaya, Mwanamamba, Tsuini and Mukuduru. In Taita Taveta county, sampling was done in Kimundia, Kiwalwa, Mwarusa and Njoro ^6^. The villages were selected based on mosquito collection data from previous studies that indicated the distribution of both *An. gambiae* and *An. funestus* complexes ^6, 30– 32^.

### Mosquito collections

In each of the sampling sites, 10 houses were randomly selected from each village. Centers for Disease Control (CDC) light traps were deployed both indoors and outdoors between 1700hrs and 0700hrs. The indoor traps were set up in a room inhabited by at least one person during that night and hung at least 2 m from the ground. The outdoor traps were set at least 5 m from the house and where possible next to a livestock enclosure. In the morning, samples were retrieved from respective traps and transported to the field laboratory in a cooler box for sorting. Additionally, indoor resting mosquitoes were aspirated using Prokopak aspirators in 30 randomly selected houses in the same villages as the CDC-light traps within Kwale county. This was done immediately after retrieval of CDC light traps in the morning and before 0700 hrs. Larval collections from natural breeding sites were also randomly done across the villages using standard dipper method and transported to the laboratory for further processing as previously described ^27^.

### Morphological identification and sample preservation

Mosquitoes were morphologically identified to species complex level using taxonomic keys ^8^. *Anopheles* mosquitoes were retained and individually dissected into different body parts: legs and wings were used for species identification by PCR; abdomens for blood meal analysis using both MALDI-TOF MS and ELISA (gold standard method); head and thorax for species identification and sporozoite detection by MALDI-TOF MS and ELISA/PCR, respectively. The Anopheline mosquitoes collected in Garithe in 2019 were first placed singly in 1.5 ml vials and preserved in silica gel for approximately 5 months before being frozen in preparation for analysis. A batch of samples collected in 2021 in Kwale were placed singly in 1.5 ml vials and kept frozen (-20°C) immediately collection and identification. Another batch of samples collected in 2022 in Taita Taveta, Kwale and Kilifi counties were placed *in silica* gel for a few days (≤ 14 days) before freezing them.

### MALDI-TOF MS analysis

***Protein extraction and plate loading*.** For species identification, either whole or half of the head and thorax were homogenised in 15 µl of 70% (v/v) formic acid (FA) (Thermo scientific, Czech Republic) and 15 µl of 50% (v/v) acetonitrile (ACN) (Thermo scientific, USA) using 106 µm acid wash glass beads (Sigma-Aldrich, USA) in a Tissue Lyser II (Qiagen, Germany) at 30 Hz for 1 min for three cycles, as previously described ^23^. For blood meal analysis, abdomens of visibly engorged mosquitoes were crushed in 50 µl LC-MS grade water (Thermo scientific, USA). A total of 10 µl of the homogenate was mixed with 10 µl of 70% FA and 10 µl of 50% ACN then mixed by vortexing. The samples were then centrifuged at 13,000 rpm for 1 min to separate the debris from the proteins. A total of 1 µl of the sample was then loaded on a MALDI-target (Bruker Daltonics) plate in quadruplicate and allowed to dry at room temperature. Thereafter, the plate was overlaid with saturated matrix solution (α-cyano-4-hydroxycynnamic acid (Sigma-Aldrich, USA), 50% (v/v) acetonitrile, 2.5% (v/v) trifluoroacetic acid (Thermo scientific, USA) and 47.5% LC-MS grade water (Thermo scientific, USA)) and again allowed to dry. Bacterial Test Standard (BTS) preparation (one spot per plate) was used as positive control and matrix only (four spots per plate) as of the negative control. The plate was then introduced into the Microflex machine (RRID:SCR_019779) (Bruker Daltonics) for spectra acquisition.

***Spectra acquisition*.** Spectra were obtained using the FlexControl software ver. 3.3.0 (Bruker Daltonics). Spectra of mass ranges 2-20 kDa were obtained in a positive linear mode at a frequency of 60 Hz, an acceleration voltage of 20 kV, extraction delay time of 200 ns, and with a maximum laser power energy of 50%. Each spectrum was based on 40 laser shots in six different regions of the sample spot.

***Spectra analysis*.** The resulting spectra were then exported to flexAnalysis (RRID:SCR_014341) ver. 3.3.0 (Bruker Daltonics) and ClinProTools ver. 4.0 (Bruker Daltonics) (free alternative, Mass-Up) for spectral cleaning and quality control and thereafter for database (DB) creation and blind testing. Spectra quality was visually checked using the FlexAnalysis ver. 3.3.0 (Bruker Daltonics) (free alternative, Mass-Up). Mass-Up software is an open-source tool for proteomics designed to support the preprocessing, analysis and classification of MALDI-TOF mass spectrometry data. Assessing general peak intensity (high intensity), the smoothness of the peaks, the flatness of baseline and its reproducibility compared to other spectra of the same categories. Only spectra of good quality were included for the subsequent analysis. Quality spectra were exported to ClinProTools ver. 4.0 (Bruker Daltonics) for principal component analysis (PCA). To further confirm quality, the spectra were loaded into MALDI-Biotyper Compass Explorer (research use only) software ver. 4.1.100. (Bruker Daltonics) (free alternative, Mass-Up) for data processing, including smoothing, baseline subtraction, normalising and peak selection. The specificity and reproducibility of the main spectrum profiles (MSPs) of different mosquito species and blood meal sources were checked by cluster analysis using MSP dendrogram and composite correlation index (CCI). MSP clustering was based on mass signals and intensities and with the expectation that mosquitoes of the same sibling species category and their blood meal source cluster on the same branch. On the other hand, CCI was done to assess spectral homogeneity/heterogeneity *i.e.*, variations within and across each sibling species and blood meal source as previously discussed ^33^. The higher the CCI value, the higher the reproducibility. Composite correlation index CCI value ranged between 0 and 1, with 0 and 1 reflecting no reproducibility and perfect reproducibility, respectively. Composite Correlation index matrix was calculated using MALDI-Biotyper Compass Explorer (research use only) software ver. 4.1.100. (Bruker Daltonics) with default settings; mass range 3.0±12.0 kDa; resolution four; eight intervals; auto-correction off.

***Database creation and blind tests (Validation)*.** The reference database containing MSPs was created for species and blood meal identification using spectral fingerprints from the cephalothorax and abdominal sections, respectively, using MALDI-Biotyper Compass Explorer (research use only) software ver. 4.1.100. (Bruker Daltonics). Spectra of good quality from each sibling species and blood meal source randomly selected were loaded in MALDI-Biotyper 3.0 software to create a reference spectra database. This was based on unprejudiced algorithm on intensity, frequency, and peak position of the MSP spectra.

Expectedly, sibling species within the *An. gambiae* and *An. funestus* complexes have high similarity in their protein signatures. Thus, to increase the discriminative power, a minimum of 10 samples per sibling species or blood meal source with high spectral reproducibility were used to create the spectral database ^18, 34^. Thereafter, unknown samples were matched against the reference database for which the software assigns a log score value (LSV) ranging from 0–3. Log score value is a biostatistical parameter that provides the level of match between the unknown sample and the reference database ^35^. Log score value ≥1.8 was considered as correct identification ^20, 36^. For *An. gambiae* sibling species identification by MALDI-TOF MS, ambiguous results (mixture of identification (among the four spots)) (Additional file 1 in *Underlying data* ^37^) can be observed as described earlier ^38^, which we also observed in the current study.

To overcome such challenges, k-nearest neighbor approach was used as previously described by Harju and colleagues ^34^, with slight modifications. Briefly, the MALDI-TOF MS identification ranking list with respective LSV was used to calculate a weighted list score to provide a summary of the list. For each sibling species identified in the ranking list, a weighted LSV was calculated by multiplying the actual LSV to the inverse of their position followed by a summing up the weighted LSV. Given that each sample was spotted in quadruplicate, the mean of the weighted LSV was calculated (Additional file 1 and Additional file 2 in *Underlying data* ^37^). As a result, the sibling species with the highest mean weighted LSV is regarded as the probable sibling species identity ^34^. This technique has been verified for species identification of closely related organisms and is being considered for use in research by Bruker Daltonics ^34^. When the top hit in at least two of the four spots per sample provided by the MALDI-Biotyper in the ranking list had discordant results with molecular approach, this technique was applied.

### Molecular identification and sequencing

Head and thorax of *An. gambiae* and *An. funestus* mosquitoes were subjected to genomic DNA extraction using Chelex protocol as previously described ^29^. A total of 5 µl of the extracts were subjected to a cocktail PCR assay employing primers targeting the intergenic spacer region (IGS) from the 5.8S and 28S coding region, and the internal transcribed region 2 (ITS2) from the 5.8S and 28S coding region flanking the variable ITS2 region for sibling species identification of *An. gambiae* and *An. funestus* complexes following the methods of Scott *et al.*, (1993) and Koekemoer *et al.*, (2002) respectively ^10, 11^. *Anopheles gambiae* complex DNA was amplified in a cocktail PCR assay ^11^ in a total reaction volume of 17 µl containing 5 µl 2X green GoTaq master mix (2X Green GoTaq® Reaction Buffer (pH 8.5), 400 μM dATP, 400 μM dGTP, 400 μM dCTP, 400 μM dTTP and 3 mM MgCl _2_), 0.5 µl of each of the five primers at a concentration of 10 Mm, 4.5 µl nuclease free water and 5 µl DNA template. The mosquito genomic DNA for *An. funestus* complex were amplified ^10^ in a total reaction volume of 17.5 µl containing 5 µl 2X green GoTaq master mix, 0.5µl of each of the six primers at a concentration of 10 Mm, 5 µl nuclease free water and 5 µl DNA template. The thermocycler conditions were one cycle of initial denaturation at 95°C for 30 sec followed by 35 cycles of denaturation, annealing and extension at 95°C for 30 min, 55°C for 45 min and 72°C for 30 sec, respectively, with a final elongation at 72°C for 10 min. Amplification products were visualised on 1.5% agarose gel stained with RedSafe™ Nucleic acid staining solution (20,000X) (iNtRON Biotechnology). *An. funestus* s.s, *An. arabiensis* and *An. gambiae* s.s DNA were used as positive controls in their respective assays whereas master mix only was used as negative control.

All samples used for MALDI-TOF MS database creation, those with equivocal results during the test run by MALDI-TOF MS, and a few randomly selected ones were subjected to sequencing using diagnostic primer targeting the ribosomal DNA ITS2 region: ITS2A (Forward primer): TGTGAACTGCAGGACACAT and ITS2B (Reverse primer): TATGCTTAAATTCAGGGGGT, as previously described ^12^. Briefly, 20 µl PCR reaction consisting of 10 µl 2X GoTaq Green Master mix (Promega Corporation, USA), 0.5 µl of each of the primers at a concentration of 10 mM was prepared and exposed to the following thermal conditions: 1 cycle of initial denaturation at 95°C followed by 35 cycles of denaturation, annealing and extension at 95°C for 2 min, 52°C for 1 min and 72°C for 30 sec, respectively, with a final elongation at 72°C for 5 min. PCR products were visualised on 1.5% agarose gel stained with Red safe staining solution. The remaining amplicons were cleaned up using ExoSAP-IT™ Express PCR Product Clean-up (Applied Biosystems; Catalogue Number 75001) as per the manufacturer’s guidelines. Lastly, the cleaned-up PCR amplicons were subjected to bi-directional Sanger sequencing as per the manufacturer’s guidelines. Briefly, the PCR reaction contained: 2 µl Nuclease free water, 1.5 µl 5X buffer, 1 µl each primer (10 mM) and 2 µl of the cleaned-up PCR amplicons/template. The PCR conditions for this assay were: 96°C for 1 min followed by 45 cycles of 96°C for 10 sec, 50°C for 5 sec and 60° for 4 min.

Using Bio-Edit (RRID:SCR_007361) software (version 7.2.5, 2013), raw ITS2 forward and reverse sequences were checked for quality, insertions, and deletions ^39^. The sequences were edited by removing primer sequences and thereafter aligned. The reverse primer sequence was reverse complimented and using the CAP contig assembly program, a contig from both reverse and forward sequences with a minimum base overlap of 20 bases and match of 85% generated. The resulting contigs were then checked for deletions and insertions and poor-quality sequences (overlapping peaks) excluded from the analysis. The resulting contigs/nucleotides were then compared to reference sequences in the National Center for Biotechnology Information (NCBI) standard nucleic acid databases using the basic local alignment search tool (BLASTN) (RRID:SCR_001598) using default search parameters.

### Blood meal analysis

Direct ELISA assay was used to discriminate blood meal sources by using affinity purified antibody phosphatase labelled goat anti-bovine IgM 0.1 mg (seraCare, USA, Cat. No: 15-12-03, Lot No.: 111264), KPL affinity purified antibody peroxidase labelled goat anti-human IgG (H+L) 1.0 mg (seraCare, USA, Cat. No.: 5220-0330; Lot No.: 10266871), KPL Peroxidase-Labelled rabbit anti-goat IgG (H+L), 0.5 mg (seraCare, USA, Cat. No. 5220-0362 (14-13-06)) and goat anti-chicken IgG (H+L) 0.5 mg (KPL, USA, Cat. No.: 14-24-06; Lot No: 150194) ^13, 14^ with slight modifications. Briefly, 1,000 µl 1X phosphate buffered saline (PBS) plain was added to the remaining homogenate crushed in 50 µl LC-MS grade as described above (protein extraction and plate loading section) then vortexed to mix. A total of 50 µl of each homogenate was loaded into a 96 well ELISA microtiter plate (Thermo scientific) and incubated at room temperature for 30 min. The plate contents were aspirated, the plate, washed, 50 µl conjugated mAbs added and plate incubated for 1 hr. The plate contents were then aspirated, plate washed 100 µl ABTS enzyme substrate (2,2'-Azinobis [3-ethylbenzothiazoline-6-sulfonic acid]-diammonium salt) and subjected to a final incubation step for 30 mins before reading the results. For each step, incubation was done at room temperature under subdued light, and washing was done three times using Tween-20 (Sigma-Aldrich, USA). Results were read visually where homogenous greenish blue colour and no colour change was considered a positive and negative result, respectively, as described previously ^14, 27^. Serum samples from human, bovine, goat, and chicken blood were used as positive controls and PBS as the negative control.

### Data analysis

Data from PCR and MALDI-TOF MS analysis was recorded and cleaned using Microsoft Excel (RRID:SCR_016137) and after that analysed using R Project for Statistical Computing (RRID:SCR_001905) Version 4.1.1 (2021-08-10) (R Core Team (2021)) ^40^. To assess the performances of MALDI-TOF MS for mosquito identification based on the storage/preservation method (fresh vs frozen vs silica gel), we compared the LSVs for each preservation method using the Kruskal-Wallis test as the data didn’t follow a Gaussian distribution.

## Results

### Morphological and molecular identity of wild-collected mosquitoes

A total of 12,038 Anophelines were collected and morphologically identified as belonging to *An. gambiae* and *An. funestus* complexes. From the entire collection, 19.37% (2332/12038) were subjected to further morphological and molecular analysis. *Anopheles gambiae* complex was composed of *An. arabiensis* (n=175), *An. merus* (n=250), *An. quadriannulatus* (n=69), unamplified (n=97), whereas *An. funestus* complex was composed of *An. rivulorum* (n=1,104), *An. funestus* (n=296), *An. leesoni* (n=27), *An. parensis* (n=7) and *An. vaneedeni* (n=4) and the rest (n=253) were unamplified ( Table 1). The samples analyzed and reported in this study is a representation of the all the sibling species of *An. gambiae* s.l and *An. funestus* s.l found in the coastal Kenya. These were sufficient to achieve the objective of this study - creating and validating the databases for species identification. We reported a few discordant results between molecular and morphological approaches. *Anopheles pretoriensis*, *An. rufipes*, *Aedes africanus*, *Anopheles cf. rivulorum* NFL-2015 and *Culex tritaeniorhynchus* were misclassified into either of the two complex species by morphology. e.g. one samples identified as *An. gambiae* s.l was identified as *An. pretoriensis*. A detailed description is in additional file 3. For purpose of MALDI-TOF MS database building, we added *An. gambiae* s.s. from an insectary colony (Kilifi strain) (n=50) because we were unable to find this species during our collections. A total of 167 samples consisting of some of the unamplified samples (n=39), those chosen for MALDI-TOF MS database creation (n=55), and a few randomly selected samples (n=73) were selected for Sanger sequencing ( Table 1). All mosquito samples used for database creation were incontrovertible. However, with the unamplified samples, results were atypical with two samples previously morphotyped as *An. gambaie* s.l. being identified as *An. pretoriensis* (Additional file 1 in *Underlying data* ^37^). Furthermore, we detected *Anopheles cf. rivulorum* NFL-2015 a new species that is for the first time reported in Kenya and has been implicated in malaria transmission in Eastern Zambia ^12^ (Additional file 3 in *Underlying data* ^37^)

**Table 1.** Summary table showing the proportion of sibling species of *An. gambiae* and *An. funestus* complexes identified using taxonomic keys and molecular methods and used for MALDI-TOF MS analysis ( *Parenthesis indicate percentages of each species/category*). Abbreviations: MALDI-TOF MS – matrix-assisted laser desorption/ionisation time-of-flight mass-spectrometry.

Species													
		*An. funestus complex*						*An. gambiae complex*					
Area of collection		*An. funestus s.s.*	*An. leesoni*	*An. parensis*	*An. rivulorum*	*An. vaneedeni*	Unamplified	*An. arabiensis*	*An. gambiae s.s.*	*An. merus*	*An. quadriannulatus*	Unamplified	Total
**County**	**Village**												
Kilifi	Burangi	0	0	0	0	0	0	15	0	1	1	5	**22**
	Garithe	0	0	0	0	0	0	69	0	232	0	6	**307**
	Insectary	0	0	0	0	0	0	0	50	0	0	0	**50**
	Marana	0	0	0	0	0	0	3	0	1	0	3	**7**
	Mtondia	0	0	0	0	0	0	15	0	0	0	0	**15**
	Sihu	6	0	0	78	0	16	0	0	0	0	0	**100**
Kwale	Fihoni	135	0	2	42	4	13	1	0	0	0	0	**197**
	Gazi	73	0	1	14	0	5	3	0	0	0	0	**96**
	Jego	0	2	0	393	0	36	40	0	0	32	70	**573**
	Kidomaya	0	0	0	33	0	2	4	0	0	15	0	**54**
	Kikwezani	0	0	1	114	0	38	0	0	1	0	0	**154**
	Kiwegu	0	0	0	24	0	5	1	0	0	0	1	**31**
	Madongoni	75	2	0	31	0	21	0	0	0	0	1	**130**
	Mangwei	0	0	0	13	0	0	1	0	1	2	0	**17**
	Marigiza	6	1	2	229	0	6	3	0	4	3	4	**258**
	Mukuduru	0	0	0	18	0	6	9	0	10	16	3	**62**
	Mwanamamba	1	0	0	1	0	1	3	0	0	0	0	**6**
	Tsuini	0	0	1	90	0	18	5	0	0	0	1	**115**
Taita Taveta	Kimundia	0	2	0	5	0	6	3	0	0	0	3	**19**
	Kiwalwa	0	4	0	1	0	48	0	0	0	0	0	**53**
	Mwarusa	0	1	0	3	0	15	0	0	0	0	0	**19**
	Njoro	0	15	0	15	0	17	0	0	0	0	0	**47**
	**Total**	**296**	**27**	**7**	**1,104**	**4**	**253**	**175**	**50**	**250**	**69**	**97**	**2,332**

### MALDI-TOF MS analysis

**Figure 1.**
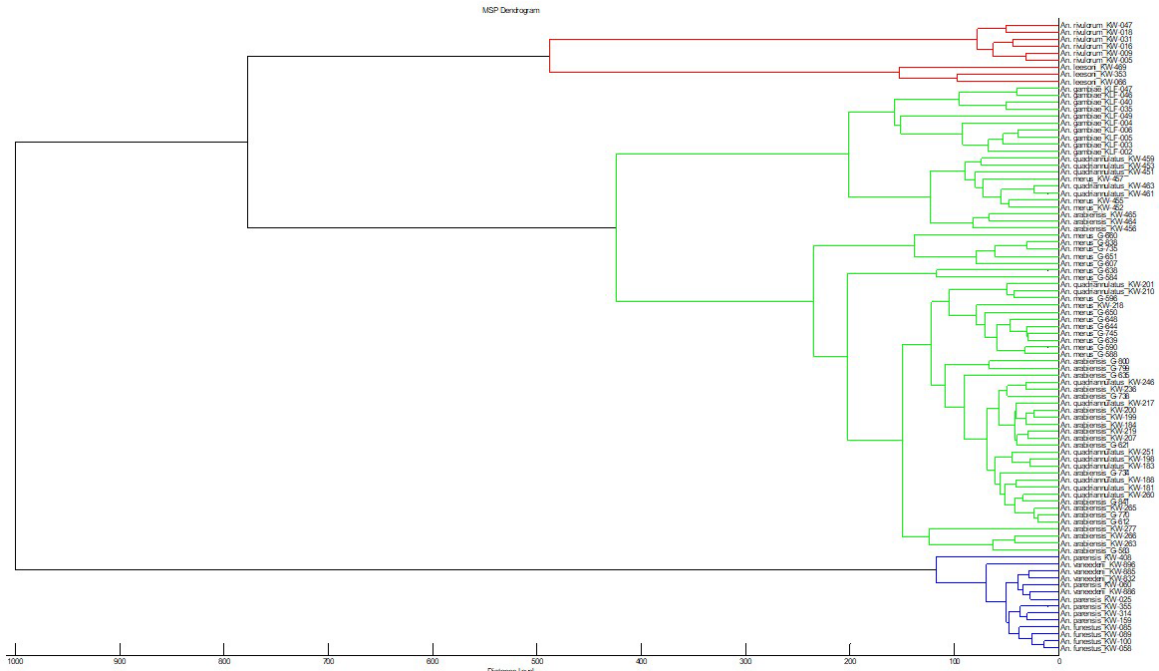
MSP dendrogram of all the MALDI-TOF MS spectra that were used for database creation for species identification as well as *An. vaneedeni* and *An. parensis.* The dendrogram was created using Biotyper v3.0 software and distance units correspond to the relative similarity of MS spectra. Abbreviations: MSP – main spectrum profile; MALDI-TOF MS – matrix-assisted laser desorption/ionization time-of-flight mass-spectrometry.

***Spectral analysis.*** Selected spectra from each sibling species in the two complexes, including *An. vaneedeni* and *An. parensis* ( Table 1), were used to perform cluster analysis to check for reproducibility and specificity using MALDI Biotyper explorer software ver. 3.3.0. We found three distinct branches: i) *An. rivulorum* and *An. leesoni* ii) *An. vaneedeni*, *An. funestus* and *An. parensis* and iii) for the *An. gambiae* complex where only *An. gambiae s.s* branched separately, while the rest of the members were mixed ( Figure 1). The reproducibility was further confirmed using CCI analyses that evaluates the relatedness of MS within and among members of *An. gambiae* and *An. funestus* complexes (Additional file 4 in *Underlying data* ^37^). Respective dark red or blue colour at the intersection square of two groups on the matrix/heat map indicates a close or incongruence relationship. Members of the respective complexes were found to be closely related; but a low correlation of MS spectra was seen between *An. gambiae* and *An. funestus* complexes (Additional file 4 in *Underlying data* ^37^) in agreement with the dendrogram ( Figure 1).

***Database creation and validation*.** Of the 2,332 mosquitoes subjected to MALDI-TOF MS, 85% (1,971/2,332) were considered for database creation and validation. The remaining 15% samples were unamplified and were not anophelines and therefore outside the scope of this current study. In the case of *An. parensis* and *An. vaneedeni* we were unable to collect sufficient specimens to allow for database creation and validation ( Table 1).

**Figure 2.**
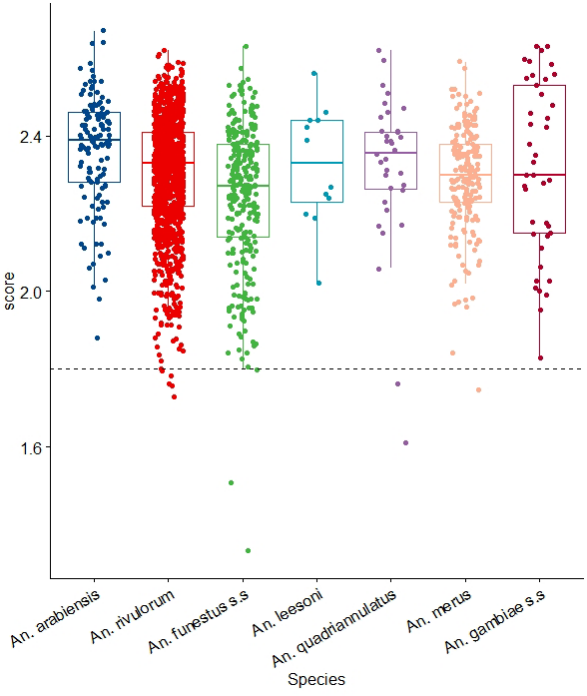
LSVs obtained after MSP reference database query with MS spectra belonging to members of *An. gambiae* and *An. funestus* complexes. Horizontal dashed lines represent the cut-off point for reliable identification (LSV > 1.8). Abbreviations: LSV – Log Score value; MSP – main spectrum profile; MS – mass-spectrometry; A.U. – arbitrary units; An. – Anopheles; s.s. – sensu stricto.

The 1,971 samples consisted of *An. gambiae* (2.5%, n=50, from insectary colony since we did not have specimens from the wild in our collections), *An. arabiensis* (8.9%, n=175), *An. merus* (12.7%, n=250), *An. quadriannulatus* (3.5%, n=69), *An. funestus* s.s. (15.0%, n=296), *An. rivulorum* (56.0%, n=1,104) and *An. leesoni* (1.4%, n=27) ( Table 2). Of the 1,971 samples, 5.2% (102/1,971) produced poor quality spectra and flatlines (suggesting that little or no ionisable proteins were present in the sample), and therefore excluded from this analysis. The remaining samples 4.4% (87/1,971) were used for database creation and 90.4% (1,782/1,971) for validation against the in-house created database. During validation, 44 samples of the 1,782 samples had a low LSV and the identities of another 26 samples were equivocal. The overall accuracy for species identification was 92.3% (1738/1884). This includes the flatlines and poor-quality spectra. However, we foresee a future, after fully optimization and really time analysis, the numbers of poor-quality spectra will be reduced or eliminated, hence will improve the overall accuracy of this technology. Considering this, 1,712/1,782 were correctly identified with an accuracy of 96.1% ( Table 2). Specifically, there was a 90.1 % and 98.3% accuracy in discriminating different members of *An. gambiae* and *An. funestus* complex, respectively ( Table 2). However, for *An. funestus*, only three sibling species were included in the database and queried ( Table 2). Overall, most of the samples, had an LSV value ≥2.00 that is above the cut-off point of 1.80 ( Figure 2). In a few scenarios, the LSV was below the 1.8 threshold, probably due to residual blood meals in the head and thorax, which could have interfered with the spectra quality.

**Table 2.** Summarises the number of samples of each sibling species that were used for MALDI-TOF MS database creation and validation as well as the accuracy of MALDI-TOF MS to identify each sibling species. Abbreviations: MALDI-TOF MS – matrix-assisted laser desorption/ionisation time-of-flight mass-spectrometry; LSV – Log score value; ID – Identification; K-NN – K-Nearest Neighbor; DB – Database.

Species	Sibling species	Total	Flatlines (Poor quality spectra)	DB creation	Validation	Low LSV	Ambiguous (Equivocal) ID	Correct ID	Accuracy (%)	Correct ID (K-NN)	Overall accuracy (%)
***An. gambiae***	*An. gambiae s. s*	50	0	10	40	0	0	40/40	100	40/40	100
	*An. arabiensis*	175	13	26	136	11	11	114/136	83.8	125/136	91.9
	*An. merus*	250	28	19	203	5	2	196/203	96.5	198/203	97.5
	*An. quadriannulatus*	69	4	14	51	5	13	33/51	64.7	46/51	90.2
***An. funestus***	*An. funestus*	296	12	6	278	16	0	262/278	94.2	262/278	94.2
	*An. rivulorum*	1104	41	6	1,057	6	0	1,051/1,057	99.4	1,051/1057	99.4
	*An. leesoni*	27	4	6	17	1	0	16/17	94.1	16/17	94.1
	**Total**	**1,971**	**102**	**87**	**1,782**	**44**	**26**	**1,712/1,782**	**96.1**	**1,738/1,782**	97.5

For the scenario where the 26 samples of the *An. gambiae* s.l., whose identities were equivocal/ambiguous, we applied a bioinformatics (K-NN) approach to discriminate among the closely related species of *An. gambiae* complex as previously described ^34^. When the top hit in at least two of the four spots per sample provided by the MALDI-Biotyper in the ranking list had discordant results with molecular results, k-nearest neighbor approach was employed (Additional file 1 and 2 in *Underlying data* ^37^). Notably, using this approach, all the samples in the *An. gambiae* complex that had equivocal/discordant results were correctly identified further improving the accuracy in species identification to 96.2% ( *An. gambiae*, 100%; *An. arabiensis*, 91.9%; *An. merus*, 97.5%; and *An. quadriannulatus*, 90.2%) ( Table 2). Thus, with this approach, the ability to discriminate between *An. funestus* and *An. gambiae* complex rose to 97.5%. To confirm the specificity of MALDI-TOF MS in species discrimination, spectra belonging to *An. pretoriensis*, *An. rufipes*, *Aedes africanus*, *Anopheles cf. rivulorum NFL-2015*, *Culex tritaeniorhynchus* (misclassified/mislabeled into either of the two complex species by morphology) were also queried against the database leading to low log score value (unidentifiable) (Additional file 3 in *Underlying data* ^37^).

### Impact of preservation method on mosquito identification and LSV distribution

**Figure 3.**
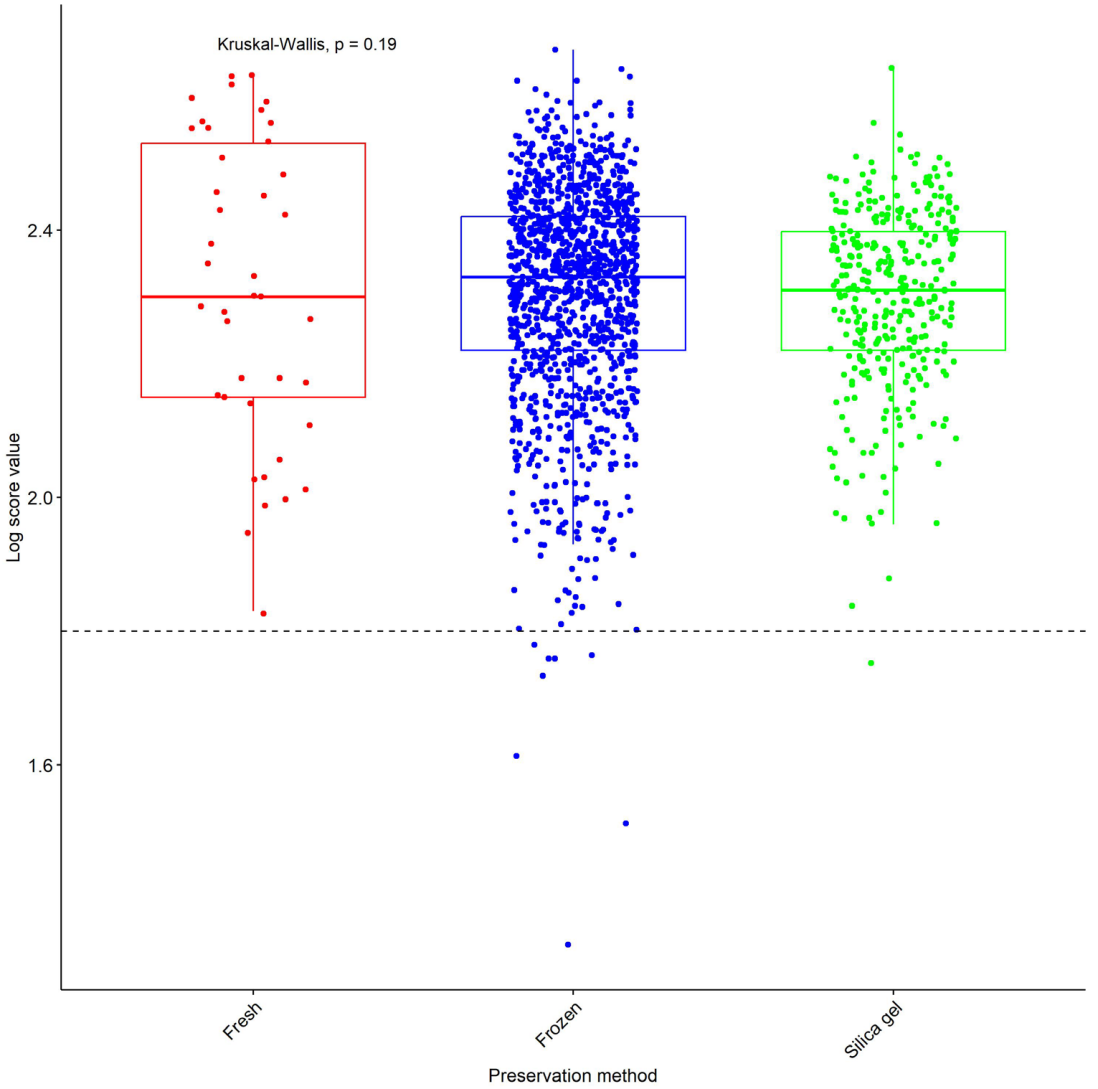
Log Score Value (LSVs) obtained after MSP reference database query with MS spectra based on method of preservation. Horizontal dashed lines represent the cut-off point for reliable identification (LSV > 1.8).

The proportion of flatlines was higher in silica gel preservation method (14.3%), followed by frozen method (4.2%) and none in the fresh samples. Moreover, the proportion low LSVs was similarly higher in silica gel preservation method (2.8%), followed by frozen method (2.1%) and none in the fresh samples. We noted that there was no significant difference (Kruskal-Wallis test, p=0.19) in the median LSV among the three methods of preservation ( Figure 3).

### Blood meal analysis by ELISA

A total of 223 mosquitoes were subjected for blood meal analysis ( Table 3). Of the 223 analysed, about half of the mosquitoes fed on bovine (53.40%; 119/223), goat (28.731%; 64/223), human (11.2%; 25/223), and multiple hosts (2.74%; 6/223) in that order whereas in a small number of samples (4.03%; 9/223) the blood meals were undetected ( Table 3). The human blood index (HBI) was 85.7% (18/21) for *An. funestus*, 23.113.6% (3/22) for *An. arabiensis*, and 4.3% (3/70) for *An. merus*. Large proportions of *An. merus* (56/70) and *An. rivulorum* (89/92) obtained their blood meals from goat and bovine, respectively ( Table 3). None of the mosquitoes fed on chicken.

**Table 3.** Proportion of blood feeding sources among the mosquitoes collected along the coastal region of Kenya ( *Parenthesis indicate percentages*).

Total Tested	Bovine	Goat	Human	Human-Goat	Bovine-Goat	N/D
	N=119	N=64	N=25	N=1	N=5	N=9
**Sibling species:**						
*An. arabiensis*	9 (7.6)	6 (9.4)	3 (12.0)	0 (0.0)	2 (40.0)	2 (22.2)
*An. funestus* s.s.	1 (0.8)	0 (0.0)	18 (72.0)	0 (0.0)	0 (0.0)	2 (22.2)
*An. leesoni*	0 (0.0)	0 (0.0)	0 (0.00)	1 (100)	0 (0.0)	0 (0.0)
*An. merus*	5 (4.2)	56 (87.5)	3 (12.0)	0 (0.0)	2 (40.0)	4 (44.4)
*An. parensis*	2 (1.7)	1 (1.56)	0 (0.0)	0 (0.0)	0 (0.0)	0 (0.0)
*An. quadriannulatus*	2 (1.7)	0 (0.00)	0 (0.0)	0 (0.0)	0 (0.0)	0 (0.0)
*An. rivulorum*	89 (74.8)	0 (0.00)	1 (4.0)	0 (0.0)	1 (20.0)	1 (11.1)
Unamplified	11 (9.24)	1 (1.56)	0 (0.0)	0 (0.0)	0 (0.0)	0 (0.0)
**Village:**						
Fihoni	2 (1.7)	1 (1.5)	10 (40.0)	0 (0.0)	0 (0.0)	2 (22.2)
Garithe	6 (5.0)	63 (98.4)	6 (24.0)	0 (0.0)	3 (60.0)	6 (66.7)
Gazi	0 (0.0)	0 (0.0)	4 (16.0)	0 (0.0)	0 (0.0)	0 (0.0)
Jego	42 (35.3)	0 (0.0)	0 (0.0)	0 (0.0)	1 (20.0)	0 (0.0)
Kidomaya	6 (5.04)	0 (0.0)	1 (4.0)	0 (0.0)	0 (0.0)	1 (11.1)
Kikwezani	3 (2.5)	0 (0.0)	0 (0.0)	0 (0.0)	0 (0.0)	0 (0.0)
Kiwegu	4 (3.4)	0 (0.0)	0 (0.0)	0 (0.0)	0 (0.0)	0 (0.0)
Madongoni	7 (5.9)	0 (0.0)	3 (12.0)	1 (100)	0 (0.0)	0 (0.0)
Mangwei	0 (0.0)	0 (0.0)	0 (0.00)	0 (0.0)	1 (20.0)	0 (0.0)
Marigiza	36 (30.3)	0 (0.0)	1 (4.0)	0 (0.0)	0 (0.0)	0 (0.0)
Mtondia	0 (0.0)	0 (0.0)	0 (0.0)	0 (0.0)	0 (0.0)	0 (0.0)
Mukuduru	10 (8.4)	0 (0.0)	0 (0.0)	0 (0.0)	0 (0.0)	0 (0.0)
Mwanamamba	3 (2.5)	0 (0.0)	0 (0.0)	0 (0.0)	0 (0.0)	0 (0.0)
**Location:**						
Indoor	18 (15.1)	3 (4.7)	19 (76.0)	0 (0.0)	2 (40.0)	4 (44.4)
Outdoor	101 (84.9)	61 (95.3)	6 (24.0)	1 (100)	3 (60.0)	5 (55.6)

### MALDI-TOF MS blood meal analysis

**Figure 4.**
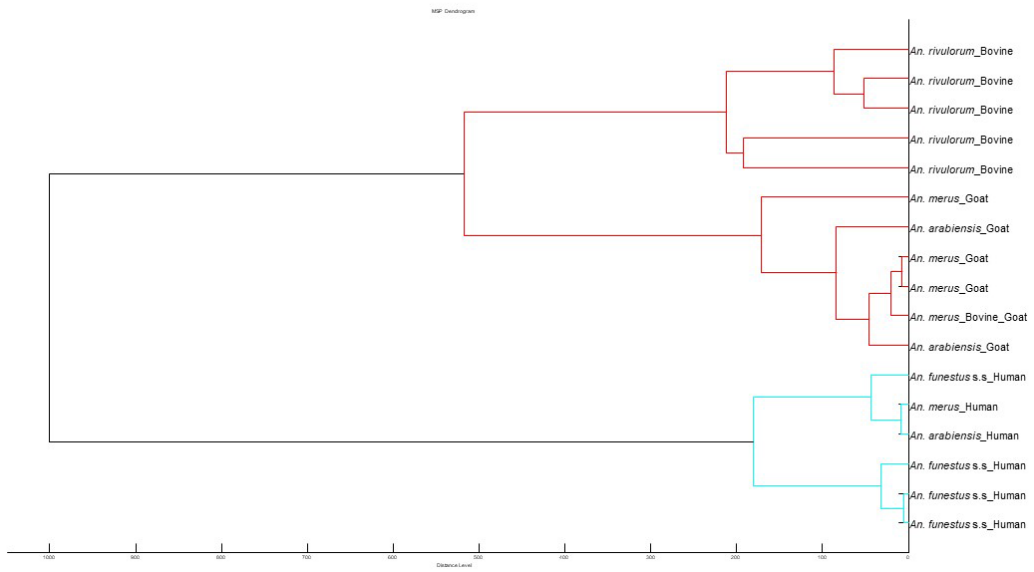
MSP dendrogram of representative MALDI-TOF MS spectra that were used for database creation for blood meal analysis identification.

All the samples 223 samples analysed by ELISA were subjected to MALDI-TOF MS for blood meal sources identification. Of the 223 samples, 201 (90%) produced high quality and reproducible spectra that were subsequently used for database creation and blind testing ( Table 4). The remaining 22 samples generated poor quality spectra or flatlines reflecting little or no proteins and were therefore excluded. Visual inspection of the spectra obtained different hosts using Flex Analysis revealed clearly distinct and highly reproducible spectral profiles within the four biological replicates spotted as well as different samples that had fed on the same host ( Figure 4A). Moreover, principal component analysis on the spectra selected for database creation classified them as distinct ( Figure 4B).

**Table 4.** Summary of blood meal sources of mosquitoes identified by ELISA and MALDI-TOF MS. Abbreviations: MALDI-TOF MS – matrix-assisted laser desorption/ionisation time-of-flight mass-spectrometry; ID – Identification; DB – Database; N/D - Not detected; N/A - Not applicable.

Sibling species	Host (ELISA)	Total	Poor quality spectra	DB Creation	Validation	Correct ID (MALDI-TOF)	Accuracy (%)
*An. arabiensis*	Bovine	9	1	0	8	8	100
	Bovine-Goat	2	1	0	1	0	0
	Goat	6	1	2	3	3	100
	Human	3	0	1	2	2	100
	N/D	2	2	N/A	N/A	N/A	N/A
*An. funestus* s.s.	Bovine	1	0	0	1	1	100
	Human	18	1	4	13	12	92.3
	N/D	2	0	N/A	N/A	N/A	N/A
*An. leesoni*	Human-Goat	1	0	0	1	0	0
*An. merus*	Bovine	5	0	1	4	3	75
	Bovine-Goat	2	0	1	1	0	0
	Goat	56	11	5	40	36	90
	Human	3	0	1	2	2	100
	N/D	4	4	0	N/A	N/A	N/A
*An. parensis*	Bovine	2	0	0	2	2	100
	Goat	1	0	0	1	1	100
*An.* *quadriannulatus*	Bovine	2	0	0	2	2	100
*An. rivulorum*	Bovine	89	0	5	84	84	100
	Bovine-Goat	1	0	0	1	0	0
	Human	1	0	0	1	0	0
	N/D	1	0	0	1	1	100
Unamplified	Bovine	11	0	0	11	11	100
	Goat	1	1	0	0	0	0
**Total**		**223**	**22**	**20**	**179**	**168**	**93.8**

**Figure 5.**
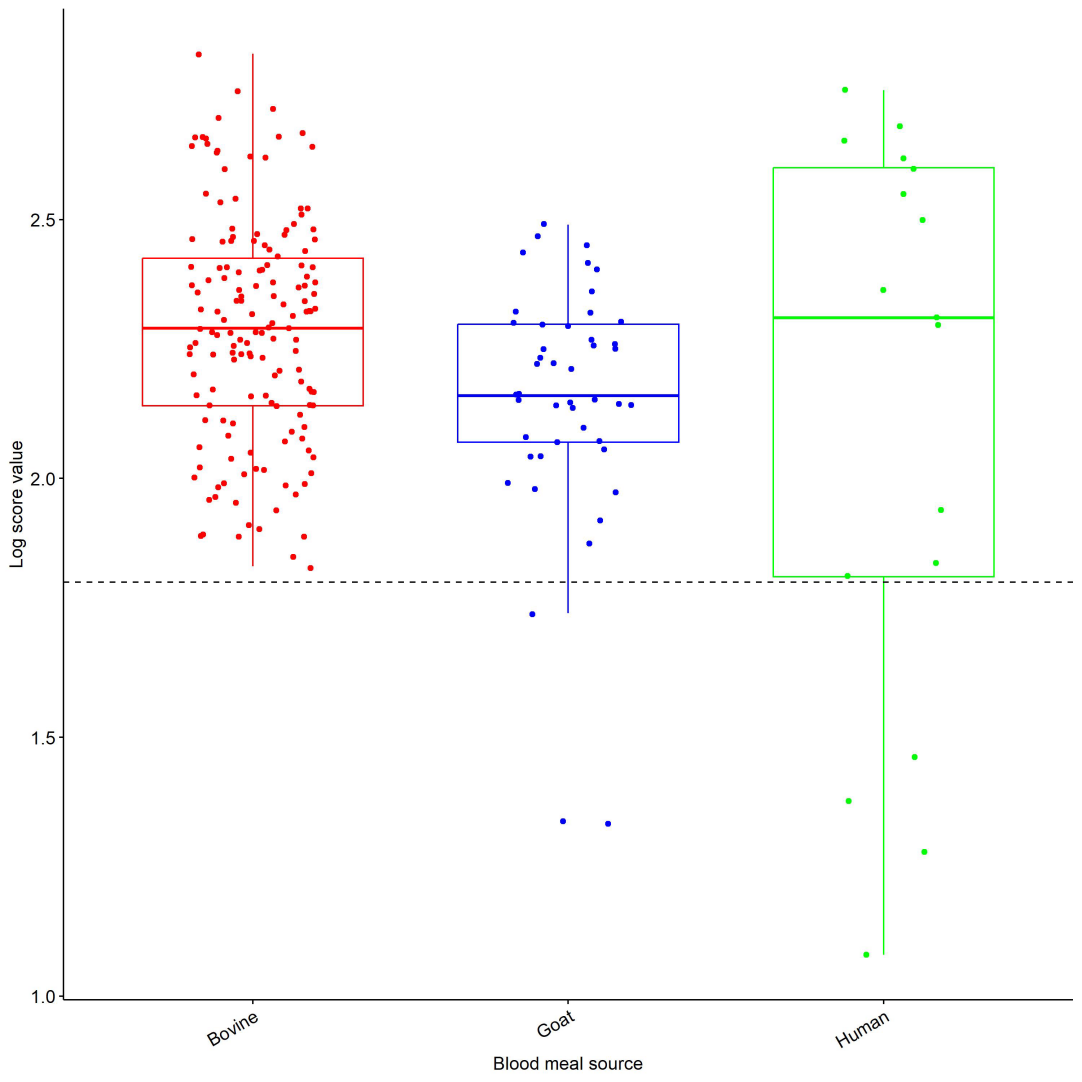
Log Score Values (LSVs) obtained during validation of the blood meal database created. Horizontal dashed lines represent the threshold value for reliable identification (LSV > 1.8).

A total of 20 different spectra were used for database creation for each different blood host. A total 201 samples were queried against the database, yielding 93.8% (168/179) correct identification ( Table 4). In the sibling species *An. funestus* s.s; two samples were not detected, hence were not included in further analysis. Similarly, in the *An. rivulorum* group, one undetected sample was not included in the analysis as the blood meal source was unknown by ELISA. Therefore, the total number of samples queried was 179 ( Table 4). During database query, there were some misclassifications in 10 samples: four goat blood meals were identified as mixed meal of bovine and goat; two Bovine-goat blood meals were classified as Bovine; one Bovine-goat mixed blood meal was classified as goat; one human blood meal was classified as bovine; one human and human-goat blood meal could not be identified. The samples (n=3) in which blood meals were inconclusive by ELISA but had quality spectra were also queried against the database, yielding LSVs of the following hosts: two bovine and one human (above the cut-off (LSV≥1.8)) ( Figure 5).

## Discussion

The study provides more information on the bionomics and diversity of the primary malaria vector and their trophic preferences along the coastal region of Kenya. This information is useful the designing targeted vector control interventions. Different sibling species belonging to *An. funestus* and *An. gambiae* complexes were reported in the region. Furthermore, the study detected *Anopheles cf. rivulorum* NFL-2015 a novel species that is for the first time reported in Kenya and has been implicated in malaria transmission in Eastern Zambia ^12^ (Additional file 3 in *Underlying data* ^37^). To be able to better our knowledge on the composition, diversity and bionomics of different vectors requires the use of cheaper, high throughput approaches.

The use of MALDI-TOF for entomological surveillance is for the first time being tested in Kenya by determining the identity of sibling species of the *An. gambiae* and *An. funestus* complexes and associated blood meal sources. To the best of our knowledge, this is the first-time members of the *An. funestus* complex have been characterised using MALDI-TOF MS. Moreover, the use of the k-nearest neighbor approach ^34^ to overcome the challenges ^38, 41^ of MALDI-TOF MS in distinguishing between members of *An. gambiae* complex has been discussed. This study further demonstrated the robustness and ability of MALDI-TOF MS in distinguishing between sibling species as well as blood meal sources of malaria vectors ^17, 38, 42, 43^. The technology was shown to efficiently discriminate members of *An. gambiae* and *An. funestus* complexes, which are the main malaria vectors in the region. The standard MALDI-TOF MS approach resulted in 96.1% (1,712/1,782) correctly identified, 1.5% (26/1,782) had ambiguous or equivocal identification and 2.5% (44/1,782) had low log score value. Misclassification could be as a result of closeness of sibling species of *An. gambiae* complex ^38, 41^, potential sample degradation or confounding proteomic changes due to mosquito’s life history, *i.e.*, physiological age progression: mating, blood feeding, oviposition ^44– 49^. Lower identification accuracy for *An. arabiensis* and *An. quadriannulatus* ( Table 2) could be due to the higher number of ambiguous or equivocal results, highlighting the closeness hence underlining the difficulty to classify specimens. In the medical microbiology sector, MALDI-TOF MS has been unable to distinguish between closely related species such as *S. pneumoniae* and other *S. mitis* species group strains ^34, 50, 51^. To control for species misidentification, k-nearest neighbor approach was developed to calculate a weighted LSV (the sum of their LSV calculated by weighted their inverse position within the ranking list) ^34^. The species with the highest summation of the weighted LSV is considered as the probable species identification. An improvement of the database and the inclusion of the algorithms used to calculate closest matches have also been suggested as means of obtaining a more reliable distinction between closely related organisms ^34^. This K-nearest neighbor technique has been verified for species identification of closely related organisms and is being considered for use in research by Bruker Daltonics in discriminating between closely related organisms. In this current study, the 26 samples that had ambiguous or equivocal identification were subjected to k-nearest neighbor method of analysis ^34^. Of note, using the weighted LSV, we were able to correctly classify all the samples that had ambiguous or equivocal identification as belonging to the *An. gambiae* complex (Additional file 2 in *Underlying data* ^37^). Interestingly, the application of k-nearest neighbor approach improved the identification accuracy of *An. gambiae* s.l. to 96.23% ( *An. gambiae* s.s – 100%, *An. arabiensis* – 91.9%, *An. merus* – 97.5%, *An. quadriannulatus* – 90.2%). The application of both standard method for results interpretation and K-nearest neighbor algorithms led to correct identification with an overall accuracy of 1,738/1,782 (97.5%).

Spectra belonging to *An. pretoriensis, An. rufipes*, *Aedes africanus*, *Anopheles cf. rivulorum NFL-2015*, *Culex tritaeniorhynchus* were also queried using the MALDI-TOF MS database and were not identifiable as they were not represented in the database, confirming the specificity of the MALDI-TOF MS. Furthermore, some specimens could not be classified and presented with LSV scores below 1.8. The inability of the method to identify these specimens could have been attributed to residual blood meals in the head and thorax ^17, 52, 53^, as well as protein degradation during shipment, storage, and processing. To improve performance of the database, inclusion spectral profiles of different physiological status and chronological ages would be helpful to improve the performance of MALDI-TOF MS in species discrimination ^17, 54^. Despite *An. parensis* and *An. vaneedeni* being reported in the molecular assays, the sample size was not enough for creation of a MALDI-TOF MS reference database and validation.

Our results confirm that the cephalothorax is well-suited for MALDI-TOF-MS database setting for identification of mosquito sibling species ^17, 42, 43^. The advantage of using the cephalothorax is that the spectra obtained may have the potential, provided databases are developed, for measuring other entomological endpoints that also reflect proteomic changes in that anatomical compartment *i.e.*, infection status ^23^ and age of mosquitoes ^55, 56^. The application of this technique in distinguishing between infected *vs.* uninfected mosquitoes has been demonstrated using laboratory-reared and artificially infected mosquitoes ^23^. Currently there are ongoing studies evaluating the applicability and robustness of the technique in determining *Plasmodium falciparum* infection status of field-collected mosquitoes as well as age-grading.

An in-depth understanding of blood meal sources of vectors provides information on the risk of vector-borne disease transmission to humans. Consistent with previous research, *An. rivulorum*, *An. parensis*, *An. quadriannulatus* and *An. merus* are highly zoophilic with a higher propensity of feeding on bovine and goats ^29, 57^. Contrary, the majority of the *An. funestus* s.s obtained their meals from human sources, further supporting the anthropophily and anthropophagy of this species ^7, 58^. Human blood meals were also detected in *An. arabiensis*, *An. merus*, *An. leesoni* and *An. rivulorum* concordant with previous studies ^7, 58^. Therefore, the role of these species as a secondary vector of *Plasmodium* species should not be overlooked.

MALDI TOF MS also accurately identified the blood meal sources irrespective of the sibling species. However, misclassifications were most likely in mixed-blood meals. Overall, there was an accuracy of 93.8% (168/179) in discriminating between different sources of blood meals. This technology showed a higher accuracy in identifying single hosts, except in a few scenarios. There were misclassifications in ten samples: four goat blood meals were identified as mixed meals of bovine-goat, and one human blood meal was classified as bovine. However, it was challenging to identify mixed meals as all the mixed meal sources were identified as single hosts. Two bovine-goat blood meals were classified as bovine; one bovine-goat mixed meal was classified as goat. These misclassifications could be attributed to insufficient sample size in the database or an imbalance in the proportions of the meals ^22^.

In some instances, this technology demonstrated superiority over the current methods where MALDI-TOF MS. could identify some samples unidentified by ELISA. For example, three samples in which blood meals were inconclusive by ELISA but had quality spectra were also queried against the database, yielding LSVs of the following hosts: two bovine and one human (above the cut-off (LSV≥1.8)). Conversely, MALDI-TOF MS also failed to classify some spectra of good quality identified as human and human-goat blood meals. This could be attributed to protein degradation or mixed meals whose origins could not be identified (a limitation of the current database). Database creation is an iterative process. It requires continued addition of spectra to capture all possible confounders that may affect identification accuracy, thereby improving the performance of MALDI-TOF MS. With comprehensive reference databases (covering all possible confounders) for species identification and blood meal analysis, it is expected that the performance of MALDI-TOF MS will improve hence revolutionizing the medical entomology sector.

## Conclusions

Accurate and reliable species identification is indispensable as it informs control programs how different vector populations are being affected by interventions. MALDI TOF MS can allow mass screening of mosquitoes, although the approach is high-tech it entails simple lab procedures, permits the processing of hundreds of samples per day, and has a very low-cost per sample. The novel approach could compliment or even replace conventional methods for mosquito species identification and blood meal determination, dramatically reducing costs and allowing surveillance programs to increase the number of samples and associated data resultant from field activities. Further research needs to be done to develop and evaluate databases for prediction of other entomological parameters of interest such as *Plasmodium* infection and age, which if successful could revolutionise entomological surveillance by creating a “silver bullet” assay whereby one test is able to inform various parameters.

## Consent for publication

All the authors have reviewed and approved the publication of this paper. This paper has been published with the permission of the Director of the Kenya Medical Research Institute (KEMRI).

## Data Availability

Harvard Dataverse: Replication Data for: Utility of MALDI-TOF MS for determination of species identity and blood meal sources of primary malaria vectors on the Kenyan coast. https://doi.org/10.7910/DVN/VYQFNO ^37^. This project contains the following underlying data: Utility of MALDI_TOF MS_Coast_dataset.tab Utility of MALDI_TOF MS_Coast_dataset_codebook.pdf Utility of MALDI_TOF MS_Coast_dataset_Readme.txt Additional file_1.pptx (Summarises the calculation of weighted Log score value using k-nearest neighbor approach) Additional file_2.docx (Show summary of the calculation of weighted LSV of a sub sample of the samples that had ambiguous/equivocal results) Additional file_3.docx (Provides a summary of sequencing results for the unamplified samples that were subjected to Sanger sequencing) Additional file_4.pptx (Assessment of *Anopheles gambiae* and *An. funestus* complex MS spectra reproducibility using composite correlation index (CCI). All the samples used for database creation in addition to *An. vaneedeni*, *An. parensis* and *An. leesoni* were subjected to analyses using the CCI tool. Levels of MS spectra reproducibility are indicated in red and blue revealing relatedness and incongruence between spectra, respectively.) Data are available under the terms of the Creative Commons Attribution 4.0 International license (CC-BY 4.0).
