# Supplementary material for: Utility of MALDI-TOF MS for determination of species identity and blood meal sources of primary malaria vectors on the Kenyan coast
Source: Wellcome Open Res. 2024 Jun 4;8:151. Originally published 2023 Mar 31. [Version 2] doi: 10.12688/wellcomeopenres.18982.2 (PMC11217722; doi:10.12688/wellcomeopenres.18982.2)
Supplement: Peer review history

# Reviewer report: Reviewer response for version 2

**DOI:** 10.21956/wellcomeopenres.22736.r85715

**Published:** 2024-07-01

**Authors:** Petr Halada

**Affiliations:** 1 Institute of Microbiology of the Czech Academy of Sciences, Prumyslova, Vestec, Czech Republic

The authors revised the manuscript extensively and improved its quality. They replied satisfactorily to the majority of my comments, however, there are still a few points remaining to address.

 Re 1:

 The haemoglobin peak might be missed in the spectra because the proteins may be degraded as the samples were preserved in silica gel at room temperature for longer periods. In our study, we observed a peak at ~7568 m/z and ~15,100 m/z in all the hosts. The results are consistent with previous research on mosquitoes freshly fed on human blood, indicating two peaks at about ~7500 m/z and 15,138 m/z (Bamou *et al.*, 2022; Reference 39). I believe the 15,138 m/z peak could represent beta-haemoglobin and a slight change in the peak could be attributed to instrument-to-instrument variations or even the sample processing procedures.

 The question on differences between ELISA and MALDI results was not answered at all. Could the authors explain why only beta-hemoglobin is degraded whereas a signal of alpha form obviously not as clearly visible in the spectra? Do the authors have any evidence that 15138 m/z corresponds to beta hemoglobin? What is the origin of m/z of 7568?

 Re 2:

 The error is well noted, and Figure 3B has been amended. The figure has been replaced with a dendrogram (now figure 4). The numbers of *An. funestus s.s*. Human-validated samples have been corrected in Table 4.

 The revised version of the manuscript is still referencing the old Fig. 4A and 4B. Should be corrected. Why, in my opinion informative and useful Fig. 3A comparing protein profiles was discarded?

 Re 3:

 We queried 179 because for *An*. *funestus* s.s; two samples were not detected and hence were not included in further analysis. Similarly, in the *An. rivulorum* group, one undetected sample was not included in the analysis as the blood meal source was unknown by ELISA.

 I still see a discrepancy in the numbers. I assume 223-22-20=181, but 179+3 = 182.

 Re 4:

 Thank you for highlighting this. I agree with you. The overall accuracy has been calculated. The overall accuracy for species identification was 92.3% (1738/1884). This includes flatlines and poor-quality spectra. However, we foresee a future, after full optimisation and real-time analysis, the numbers of poor-quality spectra will be reduced or eliminated, hence improving this technology's overall accuracy. The number of samples to be re-analysed will be negligible. This information is on page 15.

 I assume that this should be corrected in all parts of the paper (Abstract, Discussion, Table 2 etc.) and for individual Anopheles species as well.

Is the work clearly and accurately presented and does it cite the current literature?

No

If applicable, is the statistical analysis and its interpretation appropriate?

Partly

Are all the source data underlying the results available to ensure full reproducibility?

Partly

Is the study design appropriate and is the work technically sound?

Yes

Are the conclusions drawn adequately supported by the results?

Yes

Are sufficient details of methods and analysis provided to allow replication by others?

Yes

Reviewer Expertise:

Mass spectrometry of biomolecules; MALDI-TOF protein profiling; proteomics

I confirm that I have read this submission and believe that I have an appropriate level of expertise to confirm that it is of an acceptable scientific standard.

# Reviewer report: Reviewer response for version 1

**DOI:** 10.21956/wellcomeopenres.21044.r63323

**Published:** 2023-08-17

**Authors:** Petr Halada

**Affiliations:** 1 Institute of Microbiology of the Czech Academy of Sciences, Prumyslova, Vestec, Czech Republic

The work represents a comprehensive study on species and bloodmeal identification of *Anopheles *mosquitoes originating from Kenya. The paper is well-written and the methods properly described. However, there are some drawbacks, especially in the presentation of bloodmeal identification results. I have several specific comments.

 Did the authors observe any impact of storage conditions on spectrum quality (silica gel vs. direct freezing, storage time between specimen collection and MALDI-TOF MS)? Could the authors explain a significantly lower ID accuracy for *An. arabiensis* and *An. quadriannulatus* shown in the Table 2.

 Page 4: The second part of the paragraph “Spectra acquisition” is partly redundant with the paragraphs “Spectra analysis” and “DTB creation and blind tests”. The authors should rephrase those parts.

 Page 6: The Mass-Up program is mentioned. Did the authors generate any data using this SW tool? What version of MALDI BioTyper was in fact used, 3.0 or 3.3.0? Some samples gave LSV below 1.8. Did the authors find any signals related to the residual blood in the respective protein profiles?

 Did the authors see any differences between results obtained by ELISA and MALDI? In general, protein profiles from abdomen of engorged females are dominating by signals of blood hemoglobins. Could the authors comment why practically no signal of beta-hemoglobin is present in the Figure 3A.

 Some imperfections could be found between the data on bloodmeal identification presented in the Tables, Figures and the text. For example, p.6 and Table 3, how was 50% for bovine host calculated?  Page 6: “Of the 206 analysed” Why not 223? Table 3: 119 bovine hosts shown for sibling species, but 116 for village.

 There are some discrepancies between Table 4 and Figure 3B. The Table 4 shows in total three *An. merus* – Human samples and two *An. merus* – Bovine-Goat samples. However, it seems that there is four *An. merus* – Human samples (dark green) and more than two *An. merus* – Bovine-Goat samples (yellow) in Fig. 3B. Moreover, the number of *An. funestus s.s.* – Human validated samples should be corrected in the Table 4.

 Based on Table 4 and Page 8, paragraph “A total of 20 different spectra”, no mixed bloodmeals were correctly identified by MALDI-TOF MS. It is somewhat surprising that four Bovine-Goat hosts are depicted in the Figure 4. Page 8, first sentence: How was the HBI calculated for *An. arabiensis*?

 Page 8, paragraph “A total 201 sample were queried”  If 20 of 201 samples were used for DB creation, it means that 181 were subjected to MALDI for identification. However, both the following text and Table 4 works with 178 samples only.

 In addition, I suggest calculating the overall accuracy for all analysed samples including those with poor quality spectra which might truly reflect the power and efficiency of the employed approach. Is the presented MS-based methodology able to distinguish hosts having hemoglobins with almost identical masses, e.g. human and horse with alpha- and beta-hemoglobins differing by 12 and 10 Da only, respectively.

 The Discussion section is mainly devoted to mosquito species identification. The part referring to bloodmeal determination should be extended.

Is the work clearly and accurately presented and does it cite the current literature?

No

If applicable, is the statistical analysis and its interpretation appropriate?

Partly

Are all the source data underlying the results available to ensure full reproducibility?

Partly

Is the study design appropriate and is the work technically sound?

Yes

Are the conclusions drawn adequately supported by the results?

Yes

Are sufficient details of methods and analysis provided to allow replication by others?

Yes

Reviewer Expertise:

Mass spectrometry of biomolecules; MALDI-TOF protein profiling; proteomics

I confirm that I have read this submission and believe that I have an appropriate level of expertise to confirm that it is of an acceptable scientific standard, however I have significant reservations, as outlined above.

# Response

**Published:** 2023-11-17

**Authors:** Jonathan Karisa

**Affiliations:** KEMRI-Wellcome Trust Research Programme, Kenya

**Comment** **Answer**

 Major comments  

 Did the authors observe any impact of storage conditions on spectrum quality (silica gel vs. direct freezing, storage time between specimen collection and MALDI-TOF MS)? We noted that fresh and frozen samples produced good-quality spectra compared to samples stored in silica gel. Moreover, samples stored longer in silica produced more flatlines and low log score values (LSV), which could be attributable to protein degradation.

*A paragraph on the impact of storage/preservation methods on mosquito species identification has been added on page 17 and Figure 4*

 Could the authors explain a significantly lower ID accuracy for *An. arabiensis* and *An. quadriannulatus* shown in the Table 2    

*Lower ID accuracy for An. arabiensis and An. quadriannulatus could be due to their genetic closeness. The difficulties in the identification of members of An. gambiae using MALDI TOF MS has been reported elsewhere (Reference 35 (Müller et al., 2013) and reference 39 (Bamou et al., 2022)). Ambiguous results (mixture of identification (among the four spots)) were observed in the identification An. gambiae sibling species (Additional file 1). To overcome this challenge, we used the k-nearest neighbour approach, which improved the identification accuracy of An. gambiae s.l. to 96.23% (An. gambiae s.s – 100%, An. arabiensis – 91.9%, An. merus – 97.5%, An. quadriannulatus – 90.2%). This information is included in Table 2 on page 16.*

 Page 4: The second part of the paragraph "Spectra acquisition" is partly redundant with the paragraphs "Spectra analysis" and "DTB creation and blind tests". The authors should rephrase those parts.

*The paragraphs have been revised and rephrased accordingly.*

 Page 6: The Mass-Up program is mentioned. Did the authors generate any data using this SW tool? What version of MALDI BioTyper was in fact used, 3.0 or 3.3.0? Some samples gave LSV below 1.8. Did the authors find any signals related to the residual blood in the respective protein profiles?

*Only flexAnalysis software ver. 3.3.0 (Bruker Daltonics) and ClinProTools (RRID: SCR_014341) were used for spectra analysis. One of the requirements of Wellcome Open Research is to provide Research Resource Identifiers (RRID). Given that Flex Control, Flex Analysis and MALDI Biotyper Explorer are proprietary software **owned by **Bruker Daltonics, Germany, a free flexAnalysis alternative must be provided with an RRID number. That's why we settled on Mass-up software. This information is included on page 9*

 Did the authors see any differences between results obtained by ELISA and MALDI? In general, protein profiles from abdomen of engorged females are dominating by signals of blood hemoglobins. Could the authors comment why practically no signal of beta-hemoglobin is present in the Figure 3A.

*The haemoglobin peak might be missed in the spectra because the proteins may be degraded as the samples were preserved in silica gel at room temperature for longer periods. In our study, we observed a peak at ~7568 m/z and ~15,100 m/z in all the hosts. The results are consistent with previous research on mosquitoes freshly fed on human blood, indicating two peaks at about ~7500 m/z and 15,138 m/z (Bamou et al., 2022; Reference 39). I believe the 15,138 m/z peak could represent beta-haemoglobin and a slight change in the peak could be attributed to instrument-to-instrument variations or even the sample processing procedures.*

 Some imperfections could be found between the data on bloodmeal identification presented in the Tables, Figures and the text. For example, p.6 and Table 3, how was 50% for bovine host calculated?  Page 6: "Of the 206 analysed" Why not 223?

*Thank you for highlighting this. This has been corrected, and the right numbers, proportions, or percentages are included. This corrected information has been included on page 17, paragraph 1 Table 3: 119 bovine hosts shown for sibling species, but 116 for village. Thank you for highlighting this. I have rechecked, and the total number for both species and village bovine hosts detected is 119.*

 There are some discrepancies between Table 4 and Figure 3B. The Table 4 shows in total three *An. merus* – Human samples and two *An. merus* – Bovine-Goat samples. However, it seems that there is four *An. merus* – Human samples (dark green) and more than two *An. merus* – Bovine-Goat samples (yellow) in Fig. 3B. Moreover, the number of *An. funestus s.s.* – Human validated samples should be corrected in the Table 4.

*The error is well noted, and Figure 3B has been amended. The figure has been replaced with a dendrogram (now figure 4). The numbers of An. funestus s.s. – Human-validated samples have been corrected in Table 4.*

 Based on Table 4 and Page 8, paragraph "A total of 20 different spectra", no mixed bloodmeals were correctly identified by MALDI-TOF MS. It is somewhat surprising that four Bovine-Goat hosts are depicted in the Figure 4. Page 8, first sentence: How was the HBI calculated for *An. arabiensis*?

*Thank you for highlighting this. Figure 4 (now Figure 5) has been revised accordingly. Mixed meals have been removed from the graph because all mixed meals were not correctly identified. Incorrect identification could be attributed to insufficient sample size for database creation and validation or imbalance in the proportions of the meals.*

 Page 8, paragraph "A total 201 sample were queried" If 20 of 201 samples were used for DB creation, it means that 181 were subjected to MALDI for identification. However, both the following text and Table 4 works with 178 samples only.

*We queried 179 because for An. funestus s.s; two samples were not detected and hence were not included in further analysis. Similarly, in the An. rivulorum group, one undetected sample was not included in the analysis as the blood meal source was unknown by ELISA.*

 In addition, I suggest calculating the overall accuracy for all analysed samples including those with poor quality spectra which might truly reflect the power and efficiency of the employed approach.

*Thank you for highlighting this. I agree with you. The overall accuracy has been calculated. The overall accuracy for species identification was 92.3% (1738/1884). This includes flatlines and poor-quality spectra. However, we foresee a future, after full optimisation and real-time analysis, the numbers of poor-quality spectra will be reduced or eliminated, hence improving this technology's overall accuracy. The number of samples to be re-analysed will be negligible. This information is on page 15 Is the presented MS-based methodology able to distinguish hosts having hemoglobins with almost identical masses, e.g. human and horse with alpha- and beta-hemoglobins differing by 12 and 10 Da only, respectively. Yes, MALDI-TOF MS can distinguish human and horse hosts. This has been reported by Niare et al., 2016 (Reference 19).*

 The Discussion section is mainly devoted to mosquito species identification. The part referring to bloodmeal determination should be extended.

*Thank you for highlighting this. The discussion on blood meal analysis has been extended. This information is on page 23*

# Response

**Published:** 2024-02-28

**Authors:** Jonathan Karisa

**Affiliations:** KEMRI-Wellcome Trust Research Programme, Kenya

**Comment** **Answer** Major comments   Did the authors observe any impact of storage conditions on spectrum quality (silica gel vs. direct freezing, storage time between specimen collection and MALDI-TOF MS)? We noted that fresh and frozen samples produced good-quality spectra compared to samples stored in silica gel. Moreover, samples stored longer in silica produced more flatlines and low log score values (LSV), which could be attributable to protein degradation. A paragraph on the impact of storage/preservation methods on mosquito species identification has been added on page 17 and Figure 4 Could the authors explain a significantly lower ID accuracy for *An. arabiensis* and *An. quadriannulatus* shown in the Table 2 Lower ID accuracy for *An. arabiensis* and *An. quadriannulatus *could be due to their genetic closeness. The difficulties in the identification of members of *An. gambiae* using MALDI TOF MS has been reported elsewhere (Reference 35 (Müller et al., 2013) and reference 39 (Bamou et al., 2022)). Ambiguous results (mixture of identification (among the four spots)) were observed in the identification *An. gambiae* sibling species (Additional file 1). To overcome this challenge, we used the k-nearest neighbour approach, which improved the identification accuracy of *An. gambiae* s.l. to 96.23% ( *An. gambiae* s.s – 100%, *An. arabiensis* – 91.9%, *An. merus* – 97.5%, *An. quadriannulatus* – 90.2%). This information is included in Table 2 on page 16.   Page 4: The second part of the paragraph "Spectra acquisition" is partly redundant with the paragraphs "Spectra analysis" and "DTB creation and blind tests". The authors should rephrase those parts. The paragraphs have been revised and rephrased accordingly. Page 6: The Mass-Up program is mentioned. Did the authors generate any data using this SW tool? What version of MALDI BioTyper was in fact used, 3.0 or 3.3.0? Some samples gave LSV below 1.8. Did the authors find any signals related to the residual blood in the respective protein profiles? Only flexAnalysis software ver. 3.3.0 (Bruker Daltonics) and ClinProTools (RRID: SCR_014341) were used for spectra analysis. One of the requirements of Wellcome Open Research is to provide Research Resource Identifiers (RRID). Given that Flex Control, Flex Analysis and MALDI Biotyper Explorer are proprietary software **owned by **Bruker Daltonics, Germany, a free flexAnalysis alternative must be provided with an RRID number. That's why we settled on Mass-up software. This information is included on page 9 Did the authors see any differences between results obtained by ELISA and MALDI? In general, protein profiles from abdomen of engorged females are dominating by signals of blood hemoglobins. Could the authors comment why practically no signal of beta-hemoglobin is present in the Figure 3A. The haemoglobin peak might be missed in the spectra because the proteins may be degraded as the samples were preserved in silica gel at room temperature for longer periods. In our study, we observed a peak at ~7568 m/z and ~15,100 m/z in all the hosts. The results are consistent with previous research on mosquitoes freshly fed on human blood, indicating two peaks at about ~7500 m/z and 15,138 m/z (Bamou et al., 2022; Reference 39). I believe the 15,138 m/z peak could represent beta-haemoglobin and a slight change in the peak could be attributed to instrument-to-instrument variations or even the sample processing procedures. Some imperfections could be found between the data on bloodmeal identification presented in the Tables, Figures and the text. For example, p.6 and Table 3, how was 50% for bovine host calculated?  Page 6: "Of the 206 analysed" Why not 223? Thank you for highlighting this. This has been corrected, and the right numbers, proportions, or percentages are included. This corrected information has been included on page 17, paragraph 1 Table 3: 119 bovine hosts shown for sibling species, but 116 for village. Thank you for highlighting this. I have rechecked, and the total number for both species and village bovine hosts detected is 119. There are some discrepancies between Table 4 and Figure 3B. The Table 4 shows in total three *An. merus* – Human samples and two *An. merus* – Bovine-Goat samples. However, it seems that there is four *An. merus* – Human samples (dark green) and more than two *An. merus* – Bovine-Goat samples (yellow) in Fig. 3B. Moreover, the number of *An. funestus s.s.* – Human validated samples should be corrected in the Table 4. The error is well noted, and Figure 3B has been amended. The figure has been replaced with a dendrogram (now figure 4). The numbers of *An. funestus s.s.* – Human-validated samples have been corrected in Table 4.   Based on Table 4 and Page 8, paragraph "A total of 20 different spectra", no mixed bloodmeals were correctly identified by MALDI-TOF MS. It is somewhat surprising that four Bovine-Goat hosts are depicted in the Figure 4. Page 8, first sentence: How was the HBI calculated for *An. arabiensis*? Thank you for highlighting this. Figure 4 (now Figure 5) has been revised accordingly. Mixed meals have been removed from the graph because all mixed meals were not correctly identified. Incorrect identification could be attributed to insufficient sample size for database creation and validation or imbalance in the proportions of the meals. Page 8, paragraph "A total 201 sample were queried" If 20 of 201 samples were used for DB creation, it means that 181 were subjected to MALDI for identification. However, both the following text and Table 4 works with 178 samples only. We queried 179 because for *An*. *funestus* s.s; two samples were not detected and hence were not included in further analysis. Similarly, in the *An. rivulorum* group, one undetected sample was not included in the analysis as the blood meal source was unknown by ELISA. In addition, I suggest calculating the overall accuracy for all analysed samples including those with poor quality spectra which might truly reflect the power and efficiency of the employed approach. Thank you for highlighting this. I agree with you. The overall accuracy has been calculated. The overall accuracy for species identification was 92.3% (1738/1884). This includes flatlines and poor-quality spectra. However, we foresee a future, after full optimisation and real-time analysis, the numbers of poor-quality spectra will be reduced or eliminated, hence improving this technology's overall accuracy. The number of samples to be re-analysed will be negligible. This information is on page 15 Is the presented MS-based methodology able to distinguish hosts having hemoglobins with almost identical masses, e.g. human and horse with alpha- and beta-hemoglobins differing by 12 and 10 Da only, respectively. Yes, MALDI-TOF MS can distinguish human and horse hosts. This has been reported by Niare et al., 2016 (Reference 19). The Discussion section is mainly devoted to mosquito species identification. The part referring to bloodmeal determination should be extended. Thank you for highlighting this. The discussion on blood meal analysis has been extended. This information is on page 23

# Reviewer report: Reviewer response for version 1

**DOI:** 10.21956/wellcomeopenres.21044.r57657

**Published:** 2023-07-18

**Authors:** Max Maurin

**Affiliations:** 1 Universite Grenoble Alpes, Saint-Martin-d'Hères, Auvergne-Rhône-Alpes, France

This is an interesting study on the identification by MALDI TOF mass spectrometry of malaria vector mosquito species and their blood meal source in Kenya.

 The methodology and results of the study are well presented, and the manuscript is of very good quality.

 I only have a few minor comments.

 Page 6, paragraph “From the entire collection, 2332 were subjected …”. About 20% of collected mosquitoes were identified by both morphological and molecular methods. Were these samples considered representative of the whole mosquito population? Was there any discordance between morphological and molecular identifications?

 Page 6, paragraph “The remaining samples, 4.4% (87/1971) were used for database creation …”. Identification accuracy was 89.1% for *An. gambiae* complex and 98.3% for *An. funestus* complex. Although these accuracy scores are high, did the authors try to improve them by creating a database with more samples? Only 4.4% (87 samples) used for database building seems rather low to obtain accurate differentiation of such a high number of mosquito species. For closely related species belonging to the same complex, adding samples might have facilitated their differentiation.

 Different parts of mosquitoes were analyzed for molecular identification or MALDI TOF MS and for blood source characterization (legs, wing, head, thorax, and abdomen). The authors could comment on the variations in MS spectra that could be observed depending on the type of mosquito sample analyzed. What would be the most appropriate samples to use in further studies?

Is the work clearly and accurately presented and does it cite the current literature?

Yes

If applicable, is the statistical analysis and its interpretation appropriate?

Yes

Are all the source data underlying the results available to ensure full reproducibility?

Yes

Is the study design appropriate and is the work technically sound?

Yes

Are the conclusions drawn adequately supported by the results?

Yes

Are sufficient details of methods and analysis provided to allow replication by others?

Yes

Reviewer Expertise:

Clinical microbiology, bacteriology, zoonoses, arthropod-borne diseases

I confirm that I have read this submission and believe that I have an appropriate level of expertise to confirm that it is of an acceptable scientific standard.

# Response

**Published:** 2023-11-17

**Authors:** Jonathan Karisa

**Affiliations:** KEMRI-Wellcome Trust Research Programme, Kenya

**Comment** **Response**

 Comments  

 Page 6, paragraph "From the entire collection, 2332 were subjected …". About 20% of collected mosquitoes were identified by both morphological and molecular methods. Were these samples considered representative of the whole mosquito population?

*Yes, the samples analysed and reported in this study represent all the sibling species of An. gambiae s.l (An. arabiensis, An. merus & An. quadriannulatus) and An. funestus s.l (An. funestus s.s, An. rivulorum, An. leesoni, An. parensis and An. vaneedeni) complexes found in the coastal Kenya. Moreover, the samples were collected in three distinct ecological zones with different species composition viz., Kilifi, Malindi, Kwale and Taita-Taveta areas. We could analyse 19.37% (2332/12038) within the stipulated study timelines, but these were enough to create and validate the databases and represent all the sibling species of An. gambiae and An. funestus complex of interest. This information has been included on page 12 Was there any discordance between morphological and molecular identifications? We reported a few discordant results between molecular and morphological approaches. An. pretoriensis, An. rufipes, Aedes africanus, Anopheles cf. rivulorum NFL-2015, and Culex tritaeniorhynchus were misclassified into either complex species by morphology. e.g. one sample identified as An. gambiae s.l was identified as An. pretoriensis. This information has been included on page 12. A detailed description is in Additional File 3*

 Page 6, paragraph "The remaining samples, 4.4% (87/1971) were used for database creation …". Identification accuracy was 89.1% for *An. gambiae* complex and 98.3% for *An. funestus* complex. Although these accuracy scores are high, did the authors try to improve them by creating a database with more samples? Only 4.4% (87 samples) used for database building seems rather low to obtain accurate differentiation of such a high number of mosquito species. For closely related species belonging to the same complex, adding samples might have facilitated their differentiation.

*Database creation is an iterative process. It requires the continued addition of spectra to capture all possible confounders that may affect identification accuracy, thereby improving the performance of MALDI-TOF MS. However, previous studies (ref 34) have shown that ten high-quality spectra are often sufficient for database creation and calculating weighted LSV for closely related species. In this study, we used 87 sample spectra, of which 69 were from An. gambiae complex and An. funestus complex (Table 2). Therefore, these spectra were high quality and deemed sufficient for creating a robust database. This information has been included in Table 2 on page 16*

# Response

**Published:** 2024-02-28

**Authors:** Jonathan Karisa

**Affiliations:** KEMRI-Wellcome Trust Research Programme, Kenya

**Comment** **Response** Comments   Page 6, paragraph "From the entire collection, 2332 were subjected …". About 20% of collected mosquitoes were identified by both morphological and molecular methods. Were these samples considered representative of the whole mosquito population? Yes, the samples analysed and reported in this study represent all the sibling species of *An. gambiae* s.l ( *An. arabiensis*, *An. merus* & *An. quadriannulatus*) and *An. funestus *s.l ( *An. funestus* s.s, *An. rivulorum*, *An. leesoni*, *An. parensis* and *An. vaneedeni*) complexes found in the coastal Kenya. Moreover, the samples were collected in three distinct ecological zones with different species composition viz., Kilifi, Malindi, Kwale and Taita-Taveta areas. We could analyse 19.37% (2332/12038) within the stipulated study timelines, but these were enough to create and validate the databases and represent all the sibling species of *An.* *gambiae* and *An. funestus* complex of interest. This information has been included on page 12 Was there any discordance between morphological and molecular identifications? We reported a few discordant results between molecular and morphological approaches. *A* *n. pretoriensis, An. rufipes, Aedes africanus, Anopheles cf. rivulorum NFL-2015, and Culex tritaeniorhynchus* were misclassified into either complex species by morphology. e.g. one sample identified as *An. gambiae* s.l was identified as *An. pretoriensis.* This information has been included on page 12. A detailed description is in Additional File 3 Page 6, paragraph "The remaining samples, 4.4% (87/1971) were used for database creation …". Identification accuracy was 89.1% for *An. gambiae* complex and 98.3% for *An. funestus* complex. Although these accuracy scores are high, did the authors try to improve them by creating a database with more samples? Only 4.4% (87 samples) used for database building seems rather low to obtain accurate differentiation of such a high number of mosquito species. For closely related species belonging to the same complex, adding samples might have facilitated their differentiation. Database creation is an iterative process. It requires the continued addition of spectra to capture all possible confounders that may affect identification accuracy, thereby improving the performance of MALDI-TOF MS. However, previous studies (ref 34) have shown that ten high-quality spectra are often sufficient for database creation and calculating weighted LSV for closely related species. In this study, we used 87 sample spectra, of which 69 were from *An. gambiae* complex and *An. funestus* complex (Table 2). Therefore, these spectra were high quality and deemed sufficient for creating a robust database. This information has been included in Table 2 on page 16
